# A Hierarchical Bayesian Implementation of the Experience-Weighted Attraction Model

**DOI:** 10.1162/cpsy_a_00028

**Published:** 2020-11

**Authors:** Zhihao Zhang, Saksham Chandra, Andrew Kayser, Ming Hsu, Joshua L. Warren

**Affiliations:** 1 Haas School of Business, University of California, Berkeley, California, USA; 2 Social Science Matrix, University of California, Berkeley, California, USA; Department of Biostatistics, Yale University, New Haven, Connecticut, USA; 4 Helen Wills Neuroscience Institute, University of California, Berkeley, California, USA; 5 Department of Neurology, University of California, San Francisco, California, USA; 6 Department of Neurology, VA Northern California Health Care System, Mather, California, USA; 7 Haas School of Business, University of California, Berkeley, California, USA; 8 Social Science Matrix, University of California, Berkeley, California, USA; 9 Helen Wills Neuroscience Institute, University of California, Berkeley, California, USA; Department of Biostatistics, Yale University, New Haven, Connecticut, USA

**Keywords:** Bayesian hierarchical modeling, experience-weighted attraction model

## Abstract

Social and decision-making deficits are often the first symptoms of neuropsychiatric disorders. In recent years, economic games, together with computational models of strategic learning, have been increasingly applied to the characterization of individual differences in social behavior, as well as their changes across time due to disease progression, treatment, or other factors. At the same time, the high dimensionality of these data poses an important challenge to statistical estimation of these models, potentially limiting the adoption of such approaches in patients and special populations. We introduce a hierarchical Bayesian implementation of a class of strategic learning models, experience-weighted attraction (EWA), that is widely used in behavioral game theory. Importantly, this approach provides a unified framework for capturing between- and within-participant variation, including changes associated with disease progression, comorbidity, and treatment status. We show using simulated data that our hierarchical Bayesian approach outperforms representative agent and individual-level estimation methods that are commonly used in extant literature, with respect to parameter estimation and uncertainty quantification. Furthermore, using an empirical dataset, we demonstrate the value of our approach over competing methods with respect to balancing model fit and complexity. Consistent with the success of hierarchical Bayesian approaches in other areas of behavioral science, our hierarchical Bayesian EWA model represents a powerful and flexible tool to apply to a wide range of behavioral paradigms for studying the interplay between complex human behavior and biological factors.

## INTRODUCTION

Changes in social behavior and decision-making are often among the first symptoms of neuropsychiatric disorders. However, unlike disturbances in memory, emotion, or language that are readily recognized as symptoms of more serious underlying neuropsychiatric conditions, social and decision-making deficits are often overlooked. In younger patients with frontotemporal dementia, a debilitating neurodegenerative disease whose first symptoms frequently include social dysfunction, one series found a delay of greater than 6 years between symptom onset and diagnosis (Van Vliet et al., 2013). Furthermore, even when social impairments are recognized, there exist fewer behavioral measures or biomarkers to quantify such deficits, due in part to our limited knowledge of underlying neural mechanisms and their relation to mental disorders.

In recent years, there has been increasing application of economic games, together with computational models of strategic learning, to characterize social decision-making as well as its underlying neural mechanisms. For example, such computational approaches have been used to investigate the maintenance of cooperation in patients with borderline personality disorder (King-Casas et al., 2008) and the influence of depressive symptomatology on learning from social rewards (Safra, Chevallier, & Palminteri, 2019). The essence of this approach lies in the quantitative characterization of processes by which stimulus inputs drive behavioral responses, thereby enabling the use of behavioral and neural data to rigorously test existing theories of brain function and to inspire the development of new theories (O’Doherty, Hampton, & Kim, 2007).

One particularly notable example is the application of behavioral game-theoretic models to examine the biological underpinnings of strategic learning. Ample empirical evidence shows that, in a competitive game, people adapt their behavior based on both (a) rewards and punishments they receive themselves (i.e., reinforcement learning) and (b) actions of other players (i.e., belief learning). These two learning rules are formally captured by the experience-weighted attraction (EWA) model (Camerer & Ho, 1999). The EWA model provides a unified and flexible framework that allows for both effects, as well as differential weighting of them. It is a highly successful learning model that has been shown to fit and predict empirical data across a wide range of different games (Camerer, 2003; Camerer, Ho, & Chong, 2002; Galla & Farmer, 2013; Ho, Camerer, & Chong, 2007). Using this model, existing studies have identified the neural representation of key signals underlying strategic learning (Zhu, Mathewson, & Hsu, 2012), as well as effects of biological factors, such as aging (Zhu, Walsh, & Hsu, 2012), focal brain lesions (Zhu, Jiang, Scabini, Knight, & Hsu, 2019), psychiatric disorders (Hunter, Meer, Gillan, Hsu, & Daw, 2019), and genetic polymorphisms (den Ouden et al., 2013; Set et al., 2014), on learning behavior.

Despite being an attractive and versatile tool for the study of strategic learning, the EWA model can pose challenges when fit to empirical data. Because of its complexity, parameter estimation can be difficult for certain games and/or small samples (in terms of both the number of subjects and the number of rounds per subject; Camerer, 2003, section 6.8). Typically, the EWA model is fit to data from a group of subjects under the assumption that the model parameters are shared across everyone (i.e., the “representative agent” approach), using maximum-likelihood estimation. However, such an assumption of homogeneity does not necessarily hold in reality, especially in clinical populations, and it precludes the possibility of examining and explaining individual-level variability in the model parameters. More importantly, this approach prevents the investigation of the ways in which differences in model parameters may relate to demographic or biological factors or may arise from experimental interventions (e.g., pharmacological manipulations or noninvasive brain stimulation). In neglecting these questions of great significance in clinical and translational neuroscience, the “representative agent” approach has other methodological implications for the EWA model. Because of the assumption of shared parameters, the belief learning component of the model can fail to pick up idiosyncratic parameter information in individual participants’ past choice sequences (Wilcox, 2006), and the resulting group parameter fits can therefore show a misleading bias toward reinforcement learning relative to belief learning.

Some exceptions to this “representative agent” approach exist in the economics literature with certain experimental designs, allowing heterogeneity by estimating the EWA model at the individual or session level (Ho, Wang, & Camerer, 2007; Qi, Ma, Jia, & Wang, 2015). Importantly, these studies highlight that heterogeneity in the EWA model parameters is nonnegligible, consistent with results from other learning models (Cheung & Friedman, 1997). Fitting the EWA models at the individual level, however, is generally unsuitable for neuroscience studies because the small effect sizes typical of biological factors are easily buried in the relatively large noise seen in the individual-level data. In turn, this issue is often exacerbated by limited sample sizes due to practical constraints, such as the relative inaccessibility of some patient groups and the expense and time needed to recruit and study them. Furthermore, treating the participants or sessions as if they are independent may fail to fully utilize the structure of the sample (e.g., a within-subject repeated measures design) where correlations between parameters of different individuals or experimental sessions are expected and, therefore, pooling of information is appropriate.

In this article, we introduce a hierarchical Bayesian EWA model that includes participant- and session-specific model parameters, allowing for the ability to quantify variability across all parameters, to determine if there are consistent and measurable trends in the parameters as a function of participant- and session-level explanatory variables and to account for correlation that may arise from the same individual participating in multiple sessions, potentially under different sets of experimental conditions. The hierarchical framework allows for data-driven sharing of information among groups of parameters, leading to stability when attempting to estimate many parameters with potentially sparse data. In addition, our hierarchical EWA model reduces to the traditional EWA model (i.e., parameters shared across all participants) when the participant- and session-level explanatory variables are not predictive of the parameter values or no variability exists among the different groups of parameters.

Our model presents several important extensions to a recent hierarchical Bayesian analysis package (Ahn, Haines, & Zhang, 2017) that includes a specific implementation of the EWA model (for the probabilistic reversal learning task; Cools, Clark, Owen, & Robbins, 2002; den Ouden et al., 2013). Such extensions are particularly important given the increasingly complex experimental designs used in studies in computational psychiatry and related fields. First, our implementation allows for any arbitrary normal-form game structure (i.e., available strategies of both parties and the payoff matrix) and therefore can be applied to a very wide range of studies on strategic learning. It also naturally allows for the inclusion of covariates within a single framework, rather than analyzing and comparing different subgroups by repeated fittings of the model. Furthermore, our model addresses intraparticipant correlation and correlation between model parameters. Accounting for these features of the data can lead to more accurate assessment of parameter variability and improved understanding of the impact of covariates. More broadly, such an approach echoes the growing popularity of hierarchical Bayesian analyses in cognitive neuroscience and computational psychiatry (Ahn et al., 2017; Ahn, Krawitz, Kim, Busemeyer, & Brown, 2013; Ly et al., 2017; Piray, Dezfouli, Heskes, Frank, & Daw, 2019; Shiffrin, Lee, Kim, & Wagenmakers, 2008; Wiecki, Sofer, & Frank, 2013).

We test and illustrate the use of the newly developed model by examining its performance against current benchmark approaches on the patent race game (Rapoport & Amaldoss, 2000), a game-theoretic paradigm previously used in conjunction with the EWA model (Set et al., 2014; Zhu et al., 2019; Zhu, Mathewson, & Hsu, 2012; Zhu, Walsh, & Hsu, 2012), in both an empirical dataset and simulated data. In the data application, the model finds substantial evidence of a role effect (i.e., weak vs. strong), large variability in participant- and session-specific parameters, and high correlation between sets of parameters associated with the same participant. These findings highlight the need to account for such variability and correlations during modeling. The proposed model outperforms the alternative estimation approaches in providing the best balance of model fit and complexity. The advantages of the proposed model are further corroborated by a simulation study, which shows that the model has the best parameter estimation in scenarios with and without participant- and session-level variability in comparison with the existing approaches.

## METHODS

### Data Description

#### Overview

We illustrate the features of our proposed hierarchical Bayesian EWA model via an empirical dataset from a behavioral study on human participants. In this study, participants played a strategic learning game (the Patent Race game) twice, on two separate days. Importantly, the within-subject design calls for the need to capture correlation between a participant’s parameters across multiple sessions while at the same time allowing variability in the parameters across participants and sessions.

#### Participants

A total of 42 (28 female) healthy participants (i.e., without a history of neurological or psychiatric illnesses) were recruited from the University of California, Berkeley and nearby communities. All participants gave written informed consent in accordance with the Committee for the Protection of Human Subjects at the University of California, San Francisco and University of California, Berkeley. The mean age was 22.3 years (*SD* 5.9 years, range 18–47 years); ethnicity varied, including 7 Caucasian, 1 African American, 8 Hispanic, 21 Asian, and 5 mixed-ethnicity participants.

#### Procedure

During their first visit, participants underwent a medical history and physical exam. Subsequently, participants underwent two visits, during which they performed the same set of experimental tasks. At each visit, participants received task instructions for the Patent Race game and a number of other behavioral tasks. Participants underwent a functional neuroimaging session and then a behavioral session. During the latter, participants performed a series of behavioral tasks, including the Patent Race task. Only data related to the Patent Race task are reported in this article. Participants were informed that their payments would be based on one trial randomly selected from the set of choices carried out in each session of the Patent Race game as well as their performance in the other tasks.

#### Patent Race Game

**Figure 1.**
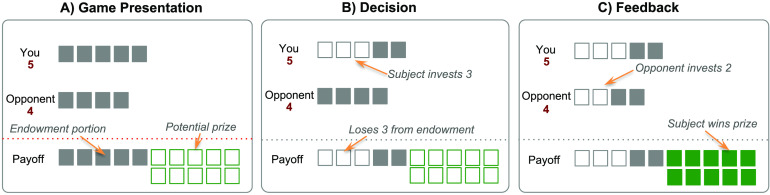
**Patent Race game**. A) After a fixation screen, subjects were presented with the Patent Race game, with information regarding their endowment, the endowment of the opponent, and the potential prize. B) Subjects input the decision (self-paced) by pressing a button mapped to the desired investment amount from the initial endowment. C) After 2–6 s, the opponent’s choice was revealed. If the subject’s investment was strictly more than those of the opponent, the subject won the prize; otherwise, the subject lost the prize. In either case, the subject kept the portion of the endowment not invested.

Prior to the experimental sessions, subjects were given instructions and completed a quiz to ensure comprehension of the game. In the Patent Race, players were matched at random at the beginning of each round and competed for a prize by choosing an investment from their respective endowments. The player who invested more won the prize, and the other lost. In the event of a tie, both lost the prize. Regardless of the outcome, players lost the amount that they invested (Figure 1). In the particular payoff structure we used, the prize was worth 10 units, and the strong (weak) player was endowed with 5 (4) units. Both players were aware of their opponent’s endowment size.

To overcome logistical difficulties of conducting simultaneous experiments with upward of 14 subjects for each behavioral subject, and to minimize unobserved session effects in opponent play associated with such a protocol, we matched subjects with choices from a pool of players who previously participated in behavioral sessions. Importantly, subjects were informed that they played in the same sequence as the pool players. That is, if the behavioral subject was playing in Round 60, the choice of opponent was drawn randomly from Round 60 of one of the pool players. Comparisons between “live” sessions and “nonlive” sessions have shown that behavior of young adults does not differ significantly across settings (Zhu, Mathewson, & Hsu, 2012).

### Hierarchical Experience-Weighted Attraction Model

Formally, the EWA model assumes that, in each round of a game, the player assigns a value (“attraction”) to each strategy in the set of possible strategies. Before the game starts, they are assumed to hold certain prior beliefs about the values of the strategies, reflecting the result of either logical deduction or previous experience. The updating of the values throughout the course of the game is then governed by three parameters, which capture qualitatively distinct aspects of the learning process, as discussed by Camerer and Ho (1999). Two parameters, *ρ* and *ϕ*, control the updating of pregame prior beliefs and of in-game experiences, respectively. The third parameter, *δ*, captures the weight between reinforcement and belief learning. The model reduces to pure reinforcement learning when *δ* = 0 and to pure belief learning when *δ* = 1. Finally, latent values are converted to choice probabilities in each round by a softmax rule, with an additional parameter *λ* that indicates the player’s sensitivity to differences in latent values or rewards. These parameters allow quantitative inferences to be made about distinct aspects of strategic learning and decision-making. Identification/estimation of these parameters also allows other learning-related latent variables to be derived (e.g., trial-by-trial prediction errors; Zhu, Mathewson, & Hsu, 2012), which is vital for characterizing the neural encoding of these signals.

We introduce the hierarchical Bayesian EWA model under the assumption that each study participant plays multiple sessions of games, possibly under different experimental conditions (but not necessarily). We denote the investment made by participant *i* during game *t* of session *j* as *Y*_*ij*_(*t*). Based on the role assigned to participant *i* during session *j*, *r*_*ij*_, he or she is free to choose from either five (weak role, *r*_*ij*_ = 0) or six (strong role, *r*_*ij*_ = 1) investment options during each game of that session. We model person *i*’s investment choice during game *t* of session *j* as a function of unknown participant- and session-specific parameters, the investments made by player *i* during the previous rounds of play (1, …, *t* − 1), and additional explanatory variables (including opponent investments during the first *t* − 1 games) such that

$$Y_{ij}(t)|\delta_{ij},\lambda_{ij},\phi_{ij},\rho_{ij},Y_{ij}(1),\ldots ,Y_{ij}\left(t-1\right)\overset{\text{ind}}{∼}\text{Categorical}\left\{p_{ij}(t)\right\}$$

for *i* = 1, …, *n* (number of participants), *j* = 1, …, *s* (number of sessions), and *t* = 1, …, *g* (number of games in each session). We make the assumption that each player participates in the same number of games in each session and the same number of sessions, but this can be relaxed without additional complexity.

The vector of probabilities corresponding to each possible investment option for player *i* during game *t* of session *j*, ***p***_*ij*_(*t*), is given as

$$p_{ij}(t)=\left\{\begin{array}{cc} {\left\{p_{ij0}(t),\ldots ,p_{ij4}(t)\right\}}^{\text{T}} & \text{if}\;r_{ij}=0\\ {\left\{p_{ij0}(t),\ldots ,p_{ij4}(t),p_{ij5}(t)\right\}}^{\text{T}} & \text{if}\;r_{ij}=1, \end{array}\right.$$

where *p*_*ijk*_(*t*) is the probability of participant *i* investing amount *k* during game *t* of session *j*. Using the standard EWA modeling framework, we define this probability such that

$$p_{ijk}(t)=\frac{\exp\left\{\lambda_{ij}V_{ijk}\left(t-1\right)\right\}}{\sum_{l=0}^{m_{ij}}\exp\left\{\lambda_{ij}V_{ijl}\left(t-1\right)\right\}},$$

where *m*_*ij*_ = 4 + *r*_*ij*_ (i.e., weak vs. strong role). The *V*_*ijk*_(*t*) function represents player *i*’s attraction of strategy *k* during session *j* after game *t* occurs. In the context of the EWA model, attractions are real numbers monotonically related to the probability of a strategy being chosen, resembling utility. As in the original EWA model (Camerer & Ho, 1999), *V*_*ijk*_(*t*) is defined recursively as

$$\frac{\phi_{ij}N_{ij}\left(t-1\right)V_{ijk}\left(t-1\right)+\left[\delta_{ij}+\left(1-\delta_{ij}\right)I\left\{Y_{ij}(t)=k\right\}\right]\pi \left\{k,Y_{ij}^{*}(t)\right\}}{N_{ij}(t)}$$

for *t* ≥ 1, where $Y_{ij}^{*}$(*t*) represents the game *t*, session *j* investment made by the opponent of player *i* (assumed known) and *I*(.) is the indicator function, which is equal to 1 when the input statement is true and to 0 otherwise. The *N*_*ij*_(*t*) function represents the “experience weight,” which is the number of “observation-equivalents” of past experience, and is also defined recursively such that

$$N_{ij}(t)=\rho_{ij}N_{ij}\left(t-1\right)+1$$

for *t* ≥ 1. Importantly, both attractions *V*_*ijk*_(*t*) and experience weights *N*_*ij*_(*t*) start with prior values *V*_*ijk*_(0) and *N*_*ij*_(0), which reflect pregame experience or belief. The *π*{*k*, $Y_{ij}^{*}$(*t*)} function represents the scalar-valued payoff that is received by participant *i* given his or her own investment and the opponent’s investment in this round and is defined such that

$$\pi \left\{k,Y_{ij}^{*}(t)\right\}=4+r_{ij}+qI\left\{k>Y_{ij}^{*}(t)\right\}-k,$$

where *q* represents the selected award amount (10 in the case of this study).

### Parameter-Level Models

The traditional EWA approach assumes that the introduced unknown model parameters are constant across participant and session such that *δ*_*ij*_ ≡ *δ* ∈ (0, 1), *λ*_*ij*_ ≡ *λ* > 0, *ϕ*_*ij*_ ≡ *ϕ* ∈ (0, 1), and *ρ*_*ij*_ ≡ *ρ* ∈ (0, 1) for all *i* and *j*. However, in our specification of the model, we allow for a more general and flexible framework that includes the original reduced form as a special case. We introduce parameter-specific regression models that aim to quantify variability in the parameters across participant and session, to determine correlation between a participant’s set of parameters across different sessions, and to characterize the associations between parameter values and participant- and session-specific explanatory variables.

The general form of the regression model (shown here for *δ*_*ij*_) is given as

$$q_{\delta }\left(\delta_{ij}\right)={\text{x}}_{i}^{\text{T}}\beta^{\delta }+{\text{z}}_{ij}^{\text{T}}\gamma^{\delta }+\theta_{i}^{\delta }+\epsilon_{ij}^{\delta },\epsilon_{ij}^{\delta }|\sigma_{\epsilon }^{2\delta }\overset{\text{iid}}{∼}\text{N}\left(0,\sigma_{\epsilon }^{2\delta }\right),$$ (1)

where *q*_*δ*_(.) is a link function that transforms the parameter to have support on the real line (i.e., *q*_*ϕ*_(.) = *q*_*δ*_(.) = *q*_*ρ*_(.) = logit(.); *q*_*λ*_(.) = ln(.)); **x**_*i*_ is a vector of explanatory variables specific to participant *i* (not changing across session), including an intercept term, and ***β***^*δ*^ is the vector of corresponding regression parameters; $\theta_{i}^{\delta }$ is the participant-level random effect that accounts for correlation between sessions played by the same person (person *i* plays *s* > 1 sessions); **z**_*ij*_ is the vector of participant- and session-specific explanatory variables (e.g., treatment status, weak/strong role) and *γ*^*δ*^ is the vector of corresponding regression parameters; and $\sigma_{\epsilon }^{2\delta }$ describes the amount of variability in the parameter values after accounting for the explanatory variables and correlation within a participant. To make group comparisons for the different parameters, additional grouping variables could be included in the **x**_*i*_ and **z**_*ij*_ vectors; posterior inference for the corresponding regression parameters would indicate whether the differences were significant.

The vector of random effect parameters specific to participant *i*, ***θ***_*i*_ = ($\theta_{i}^{\delta }$, $\theta_{i}^{\lambda }$, $\theta_{i}^{\phi }$, $\theta_{i}^{\rho }$)^T^, is modeled to account for cross correlation between the different parameters corresponding to the same participant such that ***θ***_*i*_|Σ$\overset{\text{iid}}{∼}$ MVN(**0**_4_, Σ), where MVN(**0**_4_, Σ) represents the multivariate normal distribution with mean vector equal to **0**_4_ (a vector of length 4 with all entries equal to 0) and variance/covariance matrix Σ, which describes the correlation between parameters.

### Prior Distributions

To complete the model specification, we assign weakly informative prior distributions to the introduced model hyperparameters such that $\beta_{j}^{\delta }$, $\beta_{j}^{\lambda }$, $\beta_{j}^{\phi }$, $\beta_{j}^{\rho }$ $\overset{\text{iid}}{∼}$ N(0, 100^2^), *j* = 1, …, *p*_x_, where *p*_x_ is the length of the **x**_*i*_ vector; $\gamma_{j}^{\delta }$, $\gamma_{j}^{\lambda }$, $\gamma_{j}^{\phi }$, $\gamma_{j}^{\rho }$ $\overset{\text{iid}}{∼}$ N(0, 100^2^), *j* = 1, …, *p*_z_, where *p*_z_ is the length of the **z**_*i*_ vector; $\sigma_{\epsilon }^{\delta }$, $\sigma_{\epsilon }^{\lambda }$, $\sigma_{\epsilon }^{\phi }$, $\sigma_{\epsilon }^{\rho }$ $\overset{\text{iid}}{∼}$ Uniform(0, 1000); and Σ^−1^ ∼ Wishart(*I*_4_5) (*I*_4_ represents the 4 × 4 identity matrix), allowing for marginally uniform prior correlation between each parameter (Gelman et al., 2013).

### Model Fitting

Initial values for *V*_*ijk*_(0) and *N*_*ij*_(0) are required prior to model fitting due to their recursive definitions. Owing to the added computational complexity that would be involved by treating them as free parameters to be estimated, we adopt the following simplifications in accordance with established practices in the literature (Ho, Camerer, & Chong, 2007; Zhu, Mathewson, & Hsu, 2012): (a) The *V*_*ijk*_(0) values are determined with a maximum-likelihood estimation procedure using data collected in the five initial rounds from all players sharing the same role and session and (b) *N*_*ij*_(0) are all set to 1. For the first simplification, the underlying assumption is that the initial attractions are the same among players of the same role and in the same session. Allowing different initial attractions for players of different roles is necessary because they have a different number of available strategies and different starting endowments. Because of the within-subject design, playing the game in the second session (albeit in a different role) could involve using insights from the first session to form beliefs about the attractions of the strategies. The rationale for the second simplification is that *N*_*ij*_(0) = 1 corresponds to a weak prior belief in the initial attractions in the Bayesian sense (Ho, Camerer, & Chong, 2007). This also ensures that any effect of the initial attractions *V*_*ijk*_(0) will quickly dissipate, and therefore the initialization of the attractions would not violate the general approach focusing on uncovering individual differences. We note that the estimation and subsequent specification of the initial attractions are exogenous to the hierarchical model and done prior to model fitting.

To implement the method, we take advantage of the equivalent closed forms for *N*_*ij*_(*t*) and *V*_*ijk*_(*t*), where

$$N_{ij}(t)=\rho_{ij}^{t}N_{ij}(0)+\frac{1-\rho_{ij}^{t}}{1-\rho_{ij}}$$

and

$$V_{ijk}(t)=\frac{\phi_{ij}^{t}V_{ijk}(0)N_{ij}(0)+\sum_{l=1}^{t}f_{ijk}(l)\phi_{ij}^{t-l}}{N_{ij}(t)}$$

for *t* ≥ 0, where

$$f_{ijk}(t)=\left[\delta_{ij}+\left(1-\delta_{ij}\right)I\left\{Y_{ij}(t)=k\right\}\right]\pi \left\{k,Y_{ij}^{*}(t)\right\}.$$

## DATA APPLICATION

We omit the first five games from each session for each participant to remove any effect of prior beliefs of the participants from the analyses. The participant-level explanatory variables (**x**_*i*_) include age and sex, while the participant/session-level variables (**z**_*ij*_) include the weak/strong role indicator and the session indicator (1 or 2). All models are run for 500,000 iterations, using two independently initialized chains, after removing the first 100,000 iterations as a burn-in period prior to convergence. We thin the 500,000 posterior samples by 50 to reduce posterior autocorrelation, resulting in 20,000 nearly independent posterior samples with which to make inference (10,000 from each chain). Model convergence was assessed using the Geweke diagnostic (Geweke, 1991) and visual inspection of traceplots for each model parameter. The effective sample size was calculated to ensure that an adequate number of posterior samples was collected to make accurate inference. Posterior means, standard deviations, and quantiles are presented for all parameters of interest.

**Figure 2.**
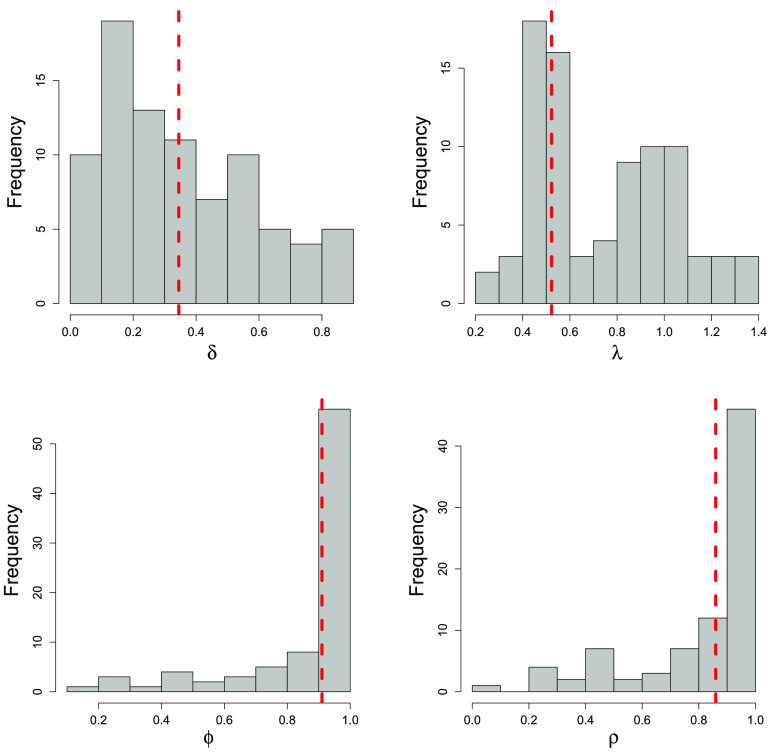
**Histogram of posterior means of the participant- and session-level parameters (*δ*_*ij*_, *λ*_*ij*_, *ϕ*_*ij*_, *ρ*_*ij*_ for all *i* and *j*) from the full model with posterior means of the parameters from the shared parameter model (*δ*, *λ*, *ϕ*, *ρ*) indicated with the red dashed line.** The average posterior standard deviations (averaged across all participants and sessions) corresponding to each grouping of parameters is *δ*: 0.12, *λ*: 0.17, *ϕ*: 0.05, *ρ*: 0.08.

In Figure 2, we present the individual- and session-specific posterior means for each set of parameters (*δ*_*ij*_, *λ*_*ij*_, *ϕ*_*ij*_, *ρ*_*ij*_ for all *i* and *j*). Clearly there is substantial variability in the parameter estimates across these factors. To explain why some of this variability is present, we present posterior inference for the different exploratory variables in Table 1. The weak/strong role indicator is consistently an important predictor of each of the parameters. Those participants in the strong role have smaller *λ*, *ϕ*, and *ρ* values on average than those in the weak role, while those in the weak role have a larger *δ* value. This indicates that players in the strong role are less sensitive to attraction differences between strategies and update their beliefs faster (i.e., more responsive to outcomes of recent rounds). With respect to differences due to session, there is no indication of a significant session effect on any parameter, as the 95% quantile-based credible intervals for all parameters include one. No other explanatory variables were shown to be important predictors of the parameters.

**Table 1.** Posterior inference for the data application presented on the odds ratio (*δ*, *ϕ*, *ρ*) and relative risk (*λ*) scales

**Effect**	**Parameter**	**Mean (*SD*)**	**Posterior quantile**		
**0.025**	**0.500**	**0.975**
Age (years)	*δ*	0.87 (0.23)	0.47	0.85	1.38
	*λ*	1.06 (0.07)	0.93	1.06	1.21
	*ϕ*	2.38 (1.93)	0.64	1.81	7.77
	*ρ*	2.25 (1.45)	0.81	1.86	6.44
Male vs. female	*δ*	1.81 (1.07)	0.57	1.58	4.41
	*λ*	0.90 (0.13)	0.66	0.89	1.19
	*ϕ*	8.15 (11.62)	0.61	4.60	39.24
	*ρ*	3.68 (3.64)	0.42	2.47	13.61
Session: 1 vs. 2	*δ*	1.15 (0.43)	0.52	1.08	2.18
	*λ*	1.16 (0.10)	0.96	1.16	1.37
	*ϕ*	0.65 (0.19)	0.36	0.63	1.08
	*ρ*	0.76 (0.22)	0.39	0.75	1.25
Strong vs. weak role	*δ*^a^	4.67 (2.18)	2.10	4.18	10.23
	*λ*^a^	0.50 (0.05)	0.42	0.50	0.60
	*ϕ*^a^	0.26 (0.08)	0.14	0.25	0.44
	*ρ*^a^	0.30 (0.09)	0.15	0.29	0.52

*Note*. ^a^95% credible interval excludes 0.

In Table 2, we display the posterior means and indicators of uncertainty for the entries of Σ, converted from raw covariances to correlations (i.e., corr_*ij*_ = Σ_*ij*_/$\sqrt{\Sigma_{ii}\Sigma_{jj}}$) for improved interpretation. Both *ϕ* and *ρ* are highly negatively correlated with *δ*, while *ϕ* and *ρ* are almost perfectly correlated. Accounting for this correlation is important during parameter estimation in order to correctly quantify uncertainty in the estimates and make accurate posterior inference.

**Table 2.** Posterior inference for the variance/covariance matrix, converted to the correlation scale

**Parameter**	***δ***	***λ***	***ϕ***	***ρ***
*δ*	1.00	−0.09	−0.65^a^	−0.68^a^
*λ*		1.00	0.07	0.16
*ϕ*			1.00	0.97^a^
*ρ*				1.00

*Note*. ^a^95% credible interval does not include zero. The credible intervals for the diagonal elements all exclude zero by definition since they are equal to one.

It is worth noting that these observations are consistent with previous studies from both empirical and theoretical standpoints. The strong correlation between *ϕ* and *ρ* likely arises from the fact that they capture common cognitive phenomena, such as forgetting and discounting old experience (Camerer & Ho, 1999). In the special case of *ϕ* = *ρ*, where the past attractions *V*_*ijk*_(*t*) and the experience measure *N*(*t*) are depreciated at the same rate, attractions will be kept in a range bounded by the game’s payoffs. The negative correlations between *ϕ* and *δ* and between *ρ* and *δ* may reflect a balance between discounting past experiences and the attention paid to other strategies (Ho, Camerer, & Chong, 2007). Intuitively, early in a game, or when there is a sudden strategy shift by the opponent, the model should put more weight on belief learning and the fictitious options (i.e., higher *δ*) and, at the same time, depreciate irrelevant past history faster (i.e., lower *ρ* and *ϕ*). On the contrary, as equilibration occurs, the model could conserve cognitive resources by attending to fewer fictitious payoffs (low *δ*) and keeping a longer window of history (high *ρ* and *ϕ*). While this notion remains to be further investigated, a recent study has revealed evidence for constraints for cognitive resources (e.g., working memory) on reinforcement learning (Collins, 2018).

## MODEL COMPARISONS

We compare the newly developed model with two competing options to provide insight into the benefits of considering variability in the parameters across person/session, sharing information across parameters during estimation, and the use of correlated random effects. To more formally compare the results from the competing models, we use a Bayesian model selection technique known as the Watanabe–Akaike information criterion (WAIC) (Watanabe, 2010). WAIC describes the performance of a model as the sum of two components, one representing model fit and one representing complexity (i.e., effective number of parameters), where smaller values of WAIC indicate better models. Preference is given to models that fit the data well while also being relatively less complex than other options. The two competing models are the (a) shared parameter model and the (b) unique participant/session parameter model. The shared parameter model represents the belief that the model parameters are shared across all participants/sessions where *δ*_*ij*_ ≡ *δ*, *λ*_*ij*_ ≡ *λ*, *ϕ*_*ij*_ ≡ *ϕ*, and *ρ*_*ij*_ ≡ *ρ* for all *i* and *j*. Therefore, no parameter-level regressions are introduced, and only four parameters are used to describe the variability in the data. While this model is a parsimonious option, we expect it to struggle greatly with respect to model fit due to a lack of flexibility and rigid assumptions regarding variability between participant- and session-level parameters.

The unique parameter model represents the opposite extreme compared to the shared parameter model where all participant- and session-level model parameters are estimated individually. In the unique parameter model, we do not include the regression framework in Equation 1 because covariates are not considered during estimation. Similarly, correlation between parameters is also ignored. While we use all of the data from every participant and session to fit the model, the parameters are specific to each participant and session and do not influence each other during estimation. We achieve this by specifying independent, weakly informative prior distributions to all model parameters such that

$$\begin{array}{c} \delta_{ij},\phi_{ij},\rho_{ij}\overset{\text{iid}}{∼}\text{Uniform}(0,1)\\ \lambda_{ij}\overset{\text{iid}}{∼}\text{Gamma}(0.01,0.01) \end{array}$$

for all *i* and *j*. This model is much more flexible than the shared parameter modelbut may result in computational problems during model fitting due to the lack of information sharing across participant- and session-level parameters. As a result, this could impact the ability of the unique parameter model to produce quality parameter estimates and measures of uncertainty. Both of the competing models are applied to the application dataset using the previously described model-fitting specifications.

### Results

The WAIC results (and effective number of parameters) for the shared parameter, unique parameter, and full models are 14,359 (6), 13,441 (318), and 13,362 (219), respectively. The shared parameter model clearly struggles to describe the variability in the data well since it uses so few parameters. The remaining models offer substantial improvements due to their increased flexibility. The full model offers the best balance between model fit and complexity among all considered models, indicating the importance of incorporating regression modeling and the cross correlation between parameters during parameter estimation.

**Figure 3.**
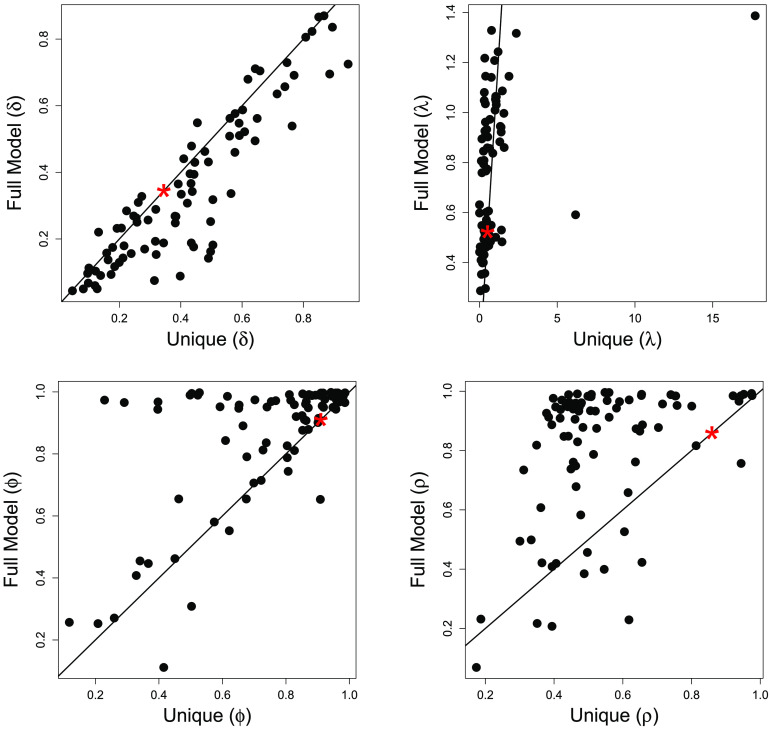
**Scatterplot of the posterior mean estimates for the participant- and session-level parameters from the full model and the unique parameter model.** The asterisk indicates the corresponding posterior mean estimated from the shared parameter model.

To further compare the competing models with the full model, we created Figure 3, which contains scatterplots of posterior mean estimates for each set of parameters (*δ*_*ij*_, *λ*_*ij*_, *ϕ*_*ij*_, and *ρ*_*ij*_ for all *i* and *j*) between the unique parameter model and the full model. Since the shared parameter model does not allow for participant- and session-specific parameters, we simply make note of the single parameter posterior mean estimate in each plot for comparison purposes. We also display the posterior mean values from the shared parameter model in Figure 2 for reference. Figure 3 suggests some large differences in parameter estimation between the unique parameter and full models, while WAIC suggests that the full model results may be most reliable.

## SIMULATED DATA

While the results on the empirical dataset strongly demonstrate the capacity of our proposed model to parsimoniously uncover heterogeneity in, and correlations between, parameters, it remains uncertain how accurate the results are, as the “true” parameters are unknown. To address this question, we simulate two datasets from the EWA model in which the model parameters are known, allowing us to more formally compare the performances of the competing models. Importantly, we leverage results from the data application described previously, so that the true parameters used in the simulations are ecologically realistic. First, we simulate data from the shared parameter model such that there are only four unknown parameters in the model. We set the values of these parameters as the posterior means obtained after fitting the shared parameter model to the application dataset. Additionally, we use other features from this dataset directly when simulating data [e.g., sample sizes, $Y_{ij}^{*}$(*t*)]. Next, we simulate data from the EWA model to include person- and session-level variability in the parameter values (i.e., four unique parameters for each participant and session combination). We use the posterior means of these parameters obtained by fitting the full model to the application dataset to set these values.

Using these two simulated datasets, we fit each of the competing models and estimate the model parameters specific to each participant and session. The model-fitting details match those from the third section, with the only difference being that one chain was used instead of two. Additionally, owing to computational constraints, only 250,000 posterior samples were collected from the unique parameter model in the analysis of the dataset, with no variability in the model parameters. For each method, we calculate the average mean absolute error (MAE) and average credible interval coverage (averaged over participants and sessions) for each set of parameters to determine which method produces estimates with reduced MAE and adequate measure of uncertainty. The average credible interval coverage is expected to be near 95% if the model is working well. We also calculate WAIC and the effective number of parameters for each model fit.

### Results

**Figure 4.**
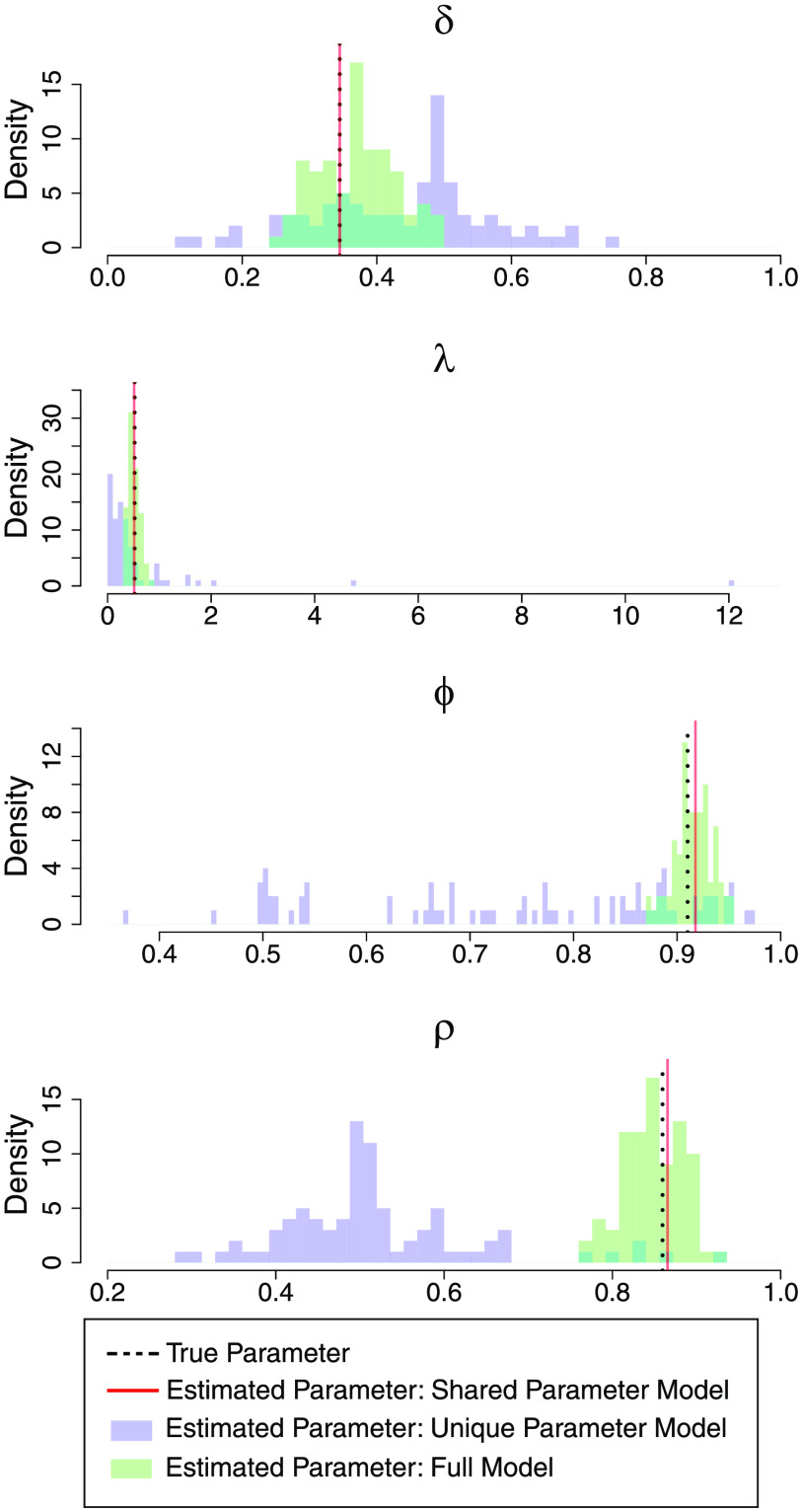
**Histograms of posterior means of the participant- and session-level parameters (*δ*_*ij*_, *λ*_*ij*_, *ϕ*_*ij*_, *ρ*_*ij*_ for all *i* and *j*) obtained from fitting the different models on the simulated dataset with no variability.** True values of the parameters for generating this simulated dataset (*δ*, *λ*, *ϕ*, *ρ*) are indicated by the black dashed lines.

**Figure 5.**
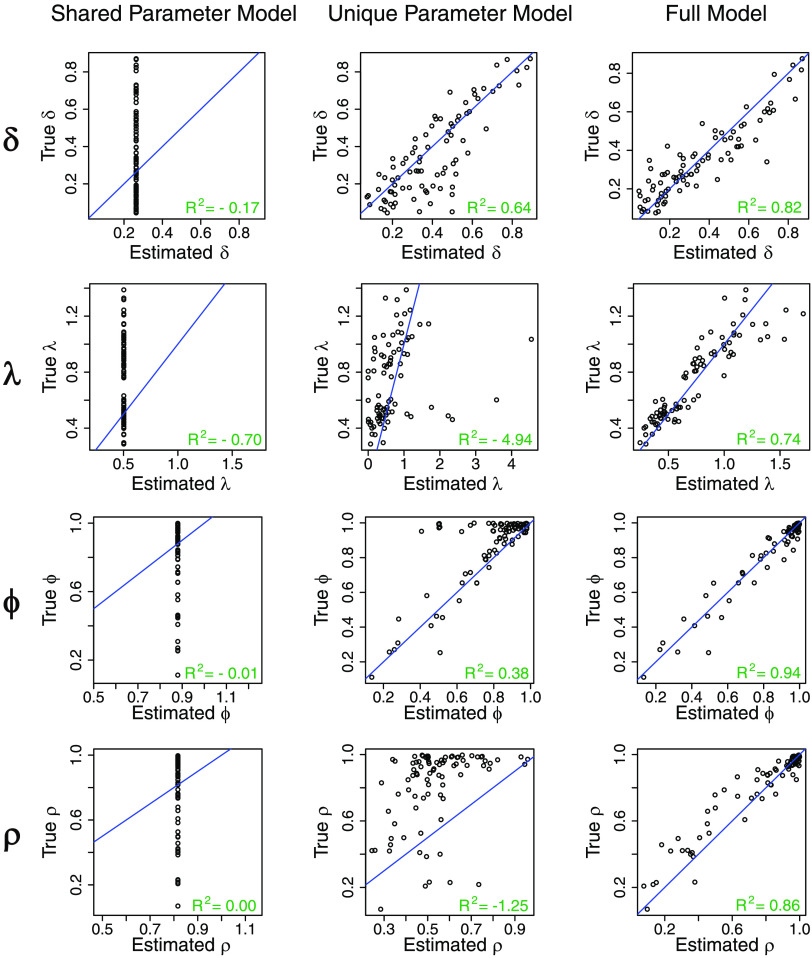
**True versus estimated (posterior means) participant- and session-level parameters (*δ*_*ij*_, *λ*_*ij*_, *ϕ*_*ij*_, *ρ*_*ij*_ for all *i* and *j*) obtained from fitting the different models on the simulated dataset with variability.** The identity line (*y* = *x*), which indicates the ideal situation where estimated parameters are exactly the same as true parameters, is shown in blue. *R*^2^ = coefficient of determination.

**Figure 6.**
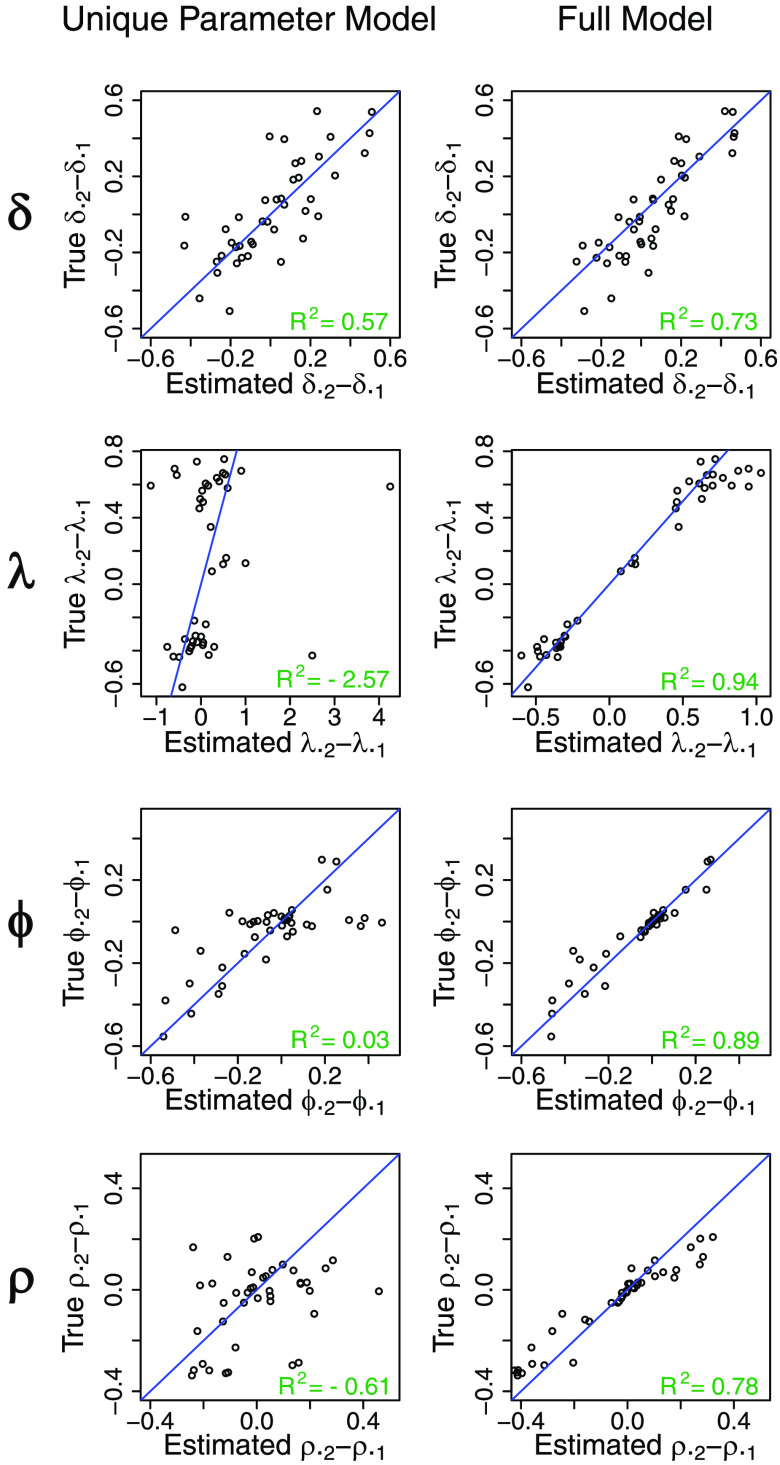
**True versus estimated session effects (i.e., differences of posterior means) on parameters (*δ*_*ij*_, *λ*_*ij*_, *ϕ*_*ij*_, *ρ*_*ij*_ for all *i*) between the two sessions obtained from fitting either the unique parameter model or the full model on the simulated dataset with variability.** The identity line (*y* = *x*), which indicates the ideal situation where estimated session effects are exactly the same as true session effects, is shown in blue. *R*^2^ = coefficient of determination.

The results are displayed in Table 3 and Figures 4–6. For the dataset without participant- and session-level variability, the full model performs well in terms of MAE with respect to the unique parameter model, while the shared parameter model (the data-generating model) is clearly best. The struggles of the unique parameter model are likely due to the estimation instability when information is not shared across sessions and parameters. For the dataset with variability in all parameters, the shared parameter model now struggles in terms of MAE and coverage across all parameters. The unique parameter model again has large MAE and low coverage for some of the parameters. The full model performs well overall, with adequate coverage and reduced MAE compared to the other models. WAIC is able to select the appropriate model for each simulated dataset. Overall, the full model performs well in both extreme settings (with and without variability), while the unique parameter model is generally not recommended in either setting.

**Table 3.** Simulation study results

**Variability**	**Model**	***δ***	***λ***	***ϕ***	***ρ***	**WAIC (EP)**
No	Shared	0.00 (1.00)	0.90 (1.00)	0.75 (1.00)	0.60 (1.00)	12,471 (4)
	Unique	13.27 (1.00)	53.84 (0.70)	17.14 (0.94)	34.02 (0.88)	13,012 (209)
	Full	5.23 (1.00)	9.61 (1.00)	1.51 (1.00)	3.01 (1.00)	12,814 (105)
Yes	Shared	19.05 (0.14)	27.74 (0.33)	14.73 (0.04)	18.16 (0.05)	12,839 (5)
	Unique	10.35 (1.00)	41.16 (0.83)	10.59 (0.83)	31.82 (0.68)	11,537 (234)
	Full	7.31 (1.00)	10.81 (0.98)	3.12 (0.95)	5.87 (0.94)	11,350 (141)

*Note.* Average mean absolute error across all participant- and session-level parameters is displayed with average coverage of the 95% credible intervals given in parentheses. Estimates of mean absolute error are multiplied by 100 for presentation purposes. EP = effective number of parameters.

We further unpack these observations by more granular visualizations of the simulation results. Figure 4 presents true versus estimated participant- and session-specific parameters by all three models of the dataset without variability. It is clear that when the true parameter does not have variability, the full model estimates fairly tight participant- and session-specific fits that are centered around the true parameter values. This indicates that the full model is able to largely reduce back to the traditional shared parameter model when little or no variability exists in the dataset and still generate accurate parameter estimates. By contrast, the unique parameter model produces fits that are not only much higher in absolute error but also heavily biased in certain cases, likely due to the instability of fitting on individual participants or sessions. Figure 5 presents the same information for the dataset with variability. Here the shared parameter model performs poorly, as expected, and the full model still outperforms the unique parameter model by being much less vulnerable to boundary fits, especially for estimation of *ϕ* and *ρ*, which again confirms the advantage of the shrinkage provided by the hierarchical approach. The superior coefficient of determination (*R*^2^) values from the full model compared with the shared parameter and unique parameter models (Figure 5) reiterate the substantial improvement in accuracy in the full model compared with the other models noted earlier.

We highlight another important feature of our model, the session effect on the parameters within participants, in Figure 6. Here we focus on the change in the same parameter in the same participant across two sessions and examine how well different models are able to recover such changes. This is a crucial test of the model, especially in the context of potential applications of this model to studies involving experimental manipulations or naturally occurring changes in behavior over time (e.g., aging or disease progression). Models that struggle in estimating the session effects will risk misidentifying such session effects. Because the shared parameter model does not allow for any variability, the comparison is focused on the full model and unique parameter model. Again, the full model performs substantially better in preserving the session effects on parameters, especially for *λ*, *ϕ*, and *ρ*.

## DISCUSSION

Using a hierarchical Bayesian EWA model permits the investigation of variability in key parameters across participant and session, the testing of possible associations between parameter values and explanatory variables, and the evaluation of correlations arising due to repeated sessions played by the same participant. Importantly, all of these factors can be flexibly adapted to reflect different specific research questions and experimental designs. The application of our proposed model can lead to a more complete understanding of how experimental factors impact game-play decisions and learning strategies, while ensuring that statistical inference is accurate. Our proposed method is completely data driven, as we generally favor weakly informative prior distributions so that our prior beliefs do not impact the final results. Of course, motivating the development of a model with the preceding characteristics is the potential for this approach to be applied to translational and clinical neuroscience studies, in which measuring and understanding individual variability are intrinsic to questions of diagnosis and treatment.

Through a within-subject multisession dataset, we have demonstrated that our proposed model outperforms the conventional representative agent approach by providing a more accurate and fine-grained fit of behavior while still minimizing model complexity. This result is achieved by fitting individual and group parameters simultaneously so that they mutually constrain each other and by incorporating cross-parameter correlations due to the within-subject nature of the study. Such an approach may offer improved sensitivity to subtle yet theoretically and practically significant effects of neurochemical agents or other interventions on learning behavior. When compared with existing modeling strategies, the hierarchical EWA model was shown to perform well with respect to model fit and complexity; it provided dramatically improved fit to the data when compared to the model that assumes all parameter values are shared across participants and sessions. In addition, inference regarding subgroups is automatically incorporated within this framework through the regression models and therefore does not require multiple fittings to different subgroups (Ahn et al., 2017). Fitting the model separately for different groups and then performing a comparison between the estimated parameters could be problematic in that it may introduce biases and false positives (Moutoussis, Hopkins, & Dolan, 2018). Similarly, in our simulation study, fitting the model separately for each individual resulted in substantial and systematic bias for parameter estimates across individuals, confounding the interpretation of these individual differences with respect to biological or psychological covariates. Our hierarchical Bayesian approach fully addresses this issue and therefore should be the preferred methodology for future studies.

Our model can be readily applied to a diverse range of research studies focused on uncovering the relationship between biological variables and behavioral signatures in strategic learning. For example, this framework is appropriate for characterizing and comparing behavioral patterns across different clinical populations—the primary goal of the emerging field of computational psychiatry (Friston, Stephan, Montague, & Dolan, 2014; Lee, 2013). In particular, impairments in social functioning have been identified as a key element of a variety of mental and psychiatric disorders (American Psychiatric Association, 2013), and novel insights into the nature of such impairments have been revealed using computational-modeling and game-theoretic approaches (Chiu et al., 2008; King-Casas et al., 2008; Yoshida, Seymour, Friston, & Dolan, 2010), including the EWA model (Crawley et al., 2019; Hunter et al., 2019). Similarly, the regression framework of our model will benefit studies examining how strategic learning behavior changes as a function of covariates of biological significance, such as age (in both developmental, Van den Bos, van Dijk, & Crone, 2012, and aging samples, Zhu, Walsh, & Hsu, 2012), symptom severity, lesion size, and drug dosage. Furthermore, our model offers more accurate derivation of individual-level latent variables during learning (Zhu, Mathewson, & Hsu, 2012). By better reflecting the underlying neurocomputational processes, improved model identification may in turn contribute to the evaluation of individual differences in the neural signatures of strategic learning through neuroimaging techniques (Daw, 2011). Additionally, the regression framework in Equation 1 can easily be extended to include any measurable factors that may explain variability between the parameters. Such factors could include additional random effects to account for more complex correlation patterns (e.g., time series correlation due to repeated measures), Bayesian variable selection, and mixture model prior distributions to induce clustering of selected variables. Specialized knowledge regarding relationships between parameters can also be incorporated by adjusting Equation 1 accordingly. However, future work considering these extensions, and others, should consider the computational complexity of the resulting models.

It is worth pointing out one caveat to the current implementation of the model. The assignment of starting values for *V*_*ijk*_(0) and *N*_*ij*_(0) could potentially have a large impact on the estimation of the unknown parameters, especially in games with smaller numbers of rounds. In earlier versions of the modeling, we attempted placing prior distributions on these parameters but encountered computational complexities arising from the introduction of many additional unknown parameters and relatively weak information in the data with which to estimate them. As a result, we chose the values using simplifying assumptions according to the relevant literature. While studies of strategic learning typically employ a relatively large number of rounds to better identify the temporal dynamics of behavior and therefore alleviate the impact of starting values, future studies may consider addressing this problem more rigorously.

Extending the proposed modeling framework, the EWA model can also be seen as a special case of model-based reinforcement learning (Doll, Bath, Daw, & Frank, 2016; Hampton, Bossaerts, & O’Doherty, 2008; Lee, Seo, & Jung, 2012), where the belief learning component is essentially a model about the opponent’s behavior. Therefore, our model could be easily generalized to model-based reinforcement learning in a nonsocial context. More broadly, through a similar hierarchical Bayesian setting, regression models could be incorporated into parameters in any computational model of behavior beyond learning. Again, the exact form of the regression model, the covariates it includes, and the correlational structure between parameters can be tailored to the specific research question of interest. The use of a multivariate normal distribution to account for cross correlation between different parameters is also applicable to non-EWA models that contain multiple unknown parameters. Common covariates in computational psychiatry studies may include group indicators for experimental manipulations (e.g., treatment vs. placebo), for disease status or severity, or for demographic characteristics (e.g., gender or age groups), as well as psychopathological or psychiatric measures (e.g., scores from psychometric scales or questionnaires).

In many settings, the hierarchical Bayesian approach provides improved estimates (i.e., reduced mean squared error) of such covariate effects, addressing challenges in accurately capturing these effects with traditional approaches, as shown by both our article and related recent work of others (e.g., Moutoussis et al., 2018). Meanwhile, accounting for intraparticipant correlation of model parameters is particularly useful in longitudinal studies or within-subject designs, which are increasingly common in computational psychiatry studies. Combined with innovative experimental designs, further applications of such models may be possible, such as transfer learning within and between tasks (Canini, Shashkov, & Griffiths, 2010). More specific theories and hypotheses about what is being transferred and what factors might affect such transfers could facilitate the formulation of more targeted treatment in the model. This would be a useful and important direction for future extensions. Accounting for correlation between different model parameters could also play an important role in gaining quantitative insights into the complex interactions between different biological and psychological processes underlying behavior. Given that the relationship between parameters intrinsic to a behavioral model and those in the regression model could be complicated, future work is needed to more specifically examine the properties of these extensions and to address any potential computational challenges.

To summarize, our hierarchical Bayesian EWA model combines state-of-the-art Bayesian modeling techniques with the well-validated EWA framework for strategic learning. It offers a number of advantages over individual- and group-level model fitting, two approaches commonly used in existing literature. In so doing, it offers a timely method, along with other newly developed approaches, such as those using the expectation-maximization algorithm (Huys et al., 2015), for addressing individual differences in behavior and their neurobiological substrates, especially as such individual differences are increasingly emphasized in basic, translational, and clinical neuroscience studies, empowering researchers to detect and dissect heterogeneity in the dynamics of learning and its biological mechanisms.

## DATA AND CODE AVAILABILITY

Simulated datasets and code for fitting the new method are available at https://github.com/warrenjl/Bayesian_EWA.

## AUTHOR CONTRIBUTIONS

Zhihao Zhang: conceptualization, data collection, analysis, writing; Saksham Chandra: analysis, methodology, writing; Andrew Kayser: conceptualization, writing; Ming Hsu: conceptualization, writing; Joshua L. Warren: analysis, methodology, writing.

## FUNDING INFORMATION

This work was partially supported by NIMH R01 MH112775 to A.K. and M.H. and DRMS 1851902 to M.H.

## ACKNOWLEDGMENTS

We thank Nikhil Kotecha, Michael Nguyen-Mason, Ekaterina Y. Goncharova, Kelsey Ichikawa, and Maxwell Good for assistance with data collection and Taylor Vega, Dawn Weinstein, and Catriona Miller for assistance with participant recruitment and screening.
